# Drug Repurposing for Identification of S1P1 Agonists with Potential Application in Multiple Sclerosis Using In Silico Drug Design Approaches

**DOI:** 10.34172/apb.2023.012

**Published:** 2022-01-03

**Authors:** Ali Akbar Alizadeh, Behzad Jafari, Siavoush Dastmalchi

**Affiliations:** ^1^Biotechnology Research Center, Tabriz University of Medical Sciences, Tabriz, Iran.; ^2^Pharmaceutical Analysis Research Center, Tabriz University of Medical Sciences, Tabriz, Iran.; ^3^Department of Medicinal Chemistry, School of Pharmacy, Urmia University of Medical Sciences, Urmia, Iran.; ^4^School of Pharmacy, Tabriz University of Medical Sciences, Tabriz, Iran.

**Keywords:** S1P agonists, Drug repurposing, Multiple sclerosis, Molecular dynamics simulations, Similarity network analysis

## Abstract

***Purpose:*** Drug repurposing is an approach successfully used for discovery of new therapeutic applications for the existing drugs. The current study was aimed to use the combination of in silico methods to identify FDA-approved drugs with possible S1P_1_ agonistic activity useful in multiple sclerosis (MS).

***Methods:*** For this, a 3D-QSAR model for the known 21 S1P_1_ agonists were generated based on 3D-QSAR approach and used to predict the possible S1P_1_ agonistic activity of FDA-approved drugs. Then, the selected compounds were screened by docking into S1P_1_ and S1P_3_ receptors to select the S1P_1_ potent and selective compounds. Further evaluation was carried out by molecular dynamics (MD) simulation studies where the S1P_1_ binding energies of selected compounds were calculated.

***Results:*** The analyses resulted in identification of cobicistat, benzonatate and brigatinib as the selective and potent S1P_1_ agonists with the binding energies of -85.93, -69.77 and -67.44 kcal. mol^-1^, calculated using MM-GBSA algorithm based on 50 ns MD simulation trajectories. These values are better than that of siponimod (-59.35 kcal mol^-1^), an FDA approved S1P_1_ agonist indicated for MS treatment. Furthermore, similarity network analysis revealed that cobicistat and brigatinib are the most structurally favorable compounds to interact with S1P_1_.

***Conclusion:*** The findings in this study revealed that cobicistat and brigatinib can be evaluated in experimental studies as potential S1P_1_ agonist candidates useful in the treatment of MS.

## Introduction

 Sphingosine 1-phosphate (S1P) is a lysophospholipid (LPL) which participates in various signaling pathways concerning immune cells development and regulation, vascular growth, cytoskeleton arrangement and morphogenesis.^1^ The signaling is initiated upon the interaction of S1P with S1P receptors (S1P_1-5_) which belong to G protein-coupled receptors family.^2^ S1P_1_ is expressed on lymphocytes (i.e., B- and T-cells) and controls immune cells trafficking.^3^ Upon activation by agonists, S1P_1_ receptor is internalized and degraded, which results in suppression of immune cells autoreactivity in target tissues.^3,4^ S1P_1_ agonists demonstrating potential for use in the treatment of autoimmune diseases have diverse structural features.^5,6^ Fingolimod (FTY720), siponimod, ponesimod and ozanimod are S1P_1_ modulators, approved by FDA for various forms of multiple sclerosis (MS), an autoimmune disorder which affect the central nervous system.^7-9^ Fingolimod is the first S1P receptor nonselective agonist developed for relapsing MS acting as a lymphocyte trafficking regulating agent.^10^ This medicine activates S1P_1_, S1P_3_, S1P_4_ and S1P_5_ receptors, while siponimod, ponesimod and ozanimod are highly selective S1P_1_ agonists.^11^ The observed adverse effects for fingolimod are correlated with its interaction with S1P_3,_^3,12^ therefore, identification of drug candidates with high S1P_1_ selectivity over S1P_3_ such as siponimod and ozanimod is of great importance in the field of drug discovery for MS treatment.^3,13,14^ In this context, several S1P modulators are in clinical trials to treat MS and other autoimmune and inflammatory disorders including Crohn’s disease, psoriasis, ulcerative colitis, and transplant rejection.^15^ In this regard, drug likeness studies are currently used for identification of drug candidates among all synthesized and naturally occurring compounds where the molecular weight, solubility and potency of FDA approved drugs are considered as guides for screening. Medicinal chemistry also offers drug repurposing of available FDA approved medications. In this strategy, structure- and ligand-based drug design techniques are applied to find new targets and therapeutic applications for already known drugs, which can be considered as a shortcut in drug discovery and development process.^16^ Various strategies are being used to achieve this goal, among which *in silico* methodologies such as pharmacophore based studies, docking techniques and molecular dynamics (MD) simulations have shown great promise. Employing this methodology bypasses the routine tedious *in vitro* and *in vivo* experiments such as ADMET (Absorption, Distribution, Metabolism, Elimination, Toxicity) studies which are cost- and time-consuming steps in the field of drug discovery and development. Identification of anti erb4 kinase activity for a sulfonamide based compound previously designed to inhibit Jumonji domain-containing protein 3 using docking studies^17^ and indinavir as an Ebola virus protease inhibitor by MD simulations^18^ are two examples of application of *in silico* procedures in drug repurposing. In the current study, a 3D-QSAR analysis using 21 S1P_1_ agonists led to the development of a model, which subsequently, was used in virtual screening of a chemical library consist of FDA-approved drugs. The selectivity of the identified compounds toward S1P_1_ and S1P_3_ was assessed by docking calculation. Finally, the receptor binding behavior of the selected therapeutics was studied through MD simulations.

## Materials and Methods

###  Generation of 3D-QSAR model for S1P_1_ agonists

 The crystal structure of S1P_1_ was retrieved from protein data bank (PDB ID:3V2Y). The 3D structures of 21 S1P_1_ agonists available in guide to pharmacology website (https://www.guidetopharmacology.org/) were downloaded from PubChem molecules database (Table 1). Using GOLD program, these 21 S1P_1_ agonists were docked into S1P_1_ according to the procedure explained in our previous work.^19^ The binding site for docking was determined based on the coordinates of an inhibitor (namely ML056) co-crystallized with the S1P_1_ (PDB ID:3V2Y). ChemPLP scoring function was used for carrying out docking process. Based on the GOLD docking scores, the best pose of each compound, was selected and used for generation of a 3D-QSAR model for S1P_1_ agonists. For this, Pentacle program, an alignment independent 3D-QSAR software was used where the 3D information of compounds were correlated with the observed S1P_1_ agonistic activities. To generate the 3D information, the compounds were introduced to Pentacle program and the corresponding molecular interaction fields (MIFs) were generated using GRID based calculations.^20^ Then, the interaction energies between each compound MIFs and the defined hydrophobic (DRY), hydrogen bond donor, HBD (O), hydrogen bond acceptor, HBA (N1) and shape (TIP) probes were calculated. The most favorable regions from MIFs were extracted using AMANDA algorithm based on the field intensity at each node of MIFs and also mutual node-node interaction distance.^21^ Finally, the maximum auto and cross-correlation (MACC2) algorithm was used for encoding the MIFs.^22^ The remnant and encoded MIFs were considered as the GRID-independent molecular descriptors (GRIND) and correlated with the experimentally determined S1P_1_ agonistic activities to generate a 3D-QSAR model. Partial least square (PLS) algorithm was used for building the 3D-QSAR model. To extract the most relevant variables, fractional factorial design method was employed. The validity of obtained final model based on 21 S1P_1_ agonists was evaluated using leave one out (LOO) and leave group out (LGO) internal cross validation methods.

**Table 1:** S1P_1_ agonists available in guide to pharmacology website (https://www.guidetopharmacology.org/) with the experimentally determined and predicted pEC_50_ values based on the generated 3D-QSAR model

**Compounds Name**	**pEC**_50 _**experimental**	**pEC**_50 _**predicted**	**Absolute error**
siponimod	9.40	9.41	0.01
SEW2871	6.50	5.78	0.72
Rp-101075	9.60	8.92	0.68
RP-001	11.10	10.18	0.92
Ponesimod	8.00	9.30	1.3
Ozanimod	9.60	9.78	0.18
Fingolimod	8.85	8.85	0
Etrasimod	8.20	8.56	0.36
CYM5442	8.90	9.15	0.25
CYM5181	8.50	7.98	0.52
Compound 43 [PMID: 26751273]	8.80	9.79	0.99
Compound 26 [PMID: 16190743]	9.20	8.50	0.7
Cenerimod	9.00	9.55	0.55
AUY954	8.90	8.53	0.37
ASP4058	8.10	7.91	0.19
Amiselimod phosphate	10.10	9.61	0.49
AFD(R)	8.60	8.97	0.37
A-971432	6.40	6.42	0.02
KRP 203-phosphate	9.10	8.09	1.01
Lysophosphatidic acid	5.60	6.99	1.39
Sphingosine 1-phosphate	8.65	9.18	0.53
Mean absolute error			0.55

###  Virtual screening

 Virtual screening was conducted to identify those FDA-approved drugs with potential ability to exert S1P_1_ agonistic activity. For this, the 3D structures of all FDA-approved drugs were downloaded from https://chemoinfo.ipmc.cnrs.fr website. These drugs were docked into the binding site of S1P_1_ and their best poses (based on the docking scores) were extracted and then their potential S1P1 agonistic activities were predicted using the 3D-QSAR model built and validated in previous section based on 21 S1P_1_ agonists. After that, the drugs were sorted based on their predicted activities and those with predicted activities higher than 8 (pEC_50 _ > 8.00) were selected. Among the selected compounds those with negative docking scores towards S1P_1_ were considered as false positives and discarded and the remnant compounds were docked into S1P_3_ using GOLD program. The S1P_3_ 3D structure was modeled based on the S1P_1_ crystal structure (PDB ID:3V2Y) as the template using modeller software from its web server (https://modbase.compbio.ucsf.edu/modweb) and its quality was checked using PROCHECK and Molprobity programs.^23,24^ ChemPLP docking scores of the selected compounds towards S1P_1_ and also the ratio of S1P_1_/ S1P_3_ docking scores were used to select the compounds with high S1P_1_ agonistic activities and selectivities.

###  Molecular dynamics simulations

 MD simulations on S1P_1_-ligand complex was carried out using AMBER suite of programs with AMBER-ff99 force field (version 14) operating on a Linux-based (Centus 6.8) GPU work station. First, CHARMM-GUI web-based platform (charmm-gui.org)^25^ was used to prepare S1P_1_-ligand complex in lipid membrane environment by embedding the complex in a hydrated, pre-equilibrated 1,2-dioleoyl-sn-glycero-3-phosphocholine (DOPC) lipid bilayer with 130 DOPC molecules per complex. Potassium and chloride ions were added at the final concentration of 150 mM to neutralize the system. Using charmmlipid2amber.py script, the obtained structure file was converted to a tleap and Lipid14 readable file. In tleap program, amber topology and initial coordinates files were produced using Lipid14^26^ and Amber-ff99SB force fields implemented in AmberTools 14. A short energy minimization was conducted on the obtained files including 5000 steps of steepest descent and 5000 steps of conjugate gradient followed by a 100 ps heating step from 0^°^K to 100^°^K in a NVT and then from 100^°^K to 303^°^K in a NPT ensembles both with 10.0 kcal.mol ^-1^. A ^-2^ harmonic restrains applied to the protein and to the lipids. Then, the system was equilibrated in the NPT ensemble at 303^°^K (controlled with Langvin thermostat) with 1 bar pressure for 2 ns followed by gradually removing the restraints. Only bond lengths involving hydrogen atoms were constrained using the SHAKE algorithm.

 The final production of dynamic simulation was performed for 50 ns by applying the Particle Mesh Ewald (PME) method under periodic boundary condition where no constraint was applied to the protein, lipids and the ligand molecules. Binding energies were calculated for ligand–receptor complexes using the molecular mechanics generalized Born surface area (MM-GBSA) algorithms. The interaction energies were calculated excluding lipid, water molecules and counter ions and presented as the average value.

###  Similarity network analysis

 In order to evaluate the similarity of selected compounds and find the most promising compounds enclosing the most essential features to interact with the target protein, similarity networks were generated for the selected ligands. At this point, top 20 compounds selected based on the predicted pEC_50_ values toward S1P_1_ were submitted into ChemBioServer.^27^ Tanimoto similarity metric was used to create a similarity matrix for the selected drugs with edge threshold set to 0.4. This matrix is generated by calcDrugFPSim function of Rcpi package which calculates the drug molecules’ similarity derived from their molecular fingerprints.^28^ Cytoscape software^29^ was used to visualize the similarity networks. The hub objects were identified using cytoHubba application of Cytoscape where the objects were ranked based on degree of correlation for each compound. At the same time, the structures were clustered based on the Tanimoto values using edge-weighted spring embedded layout.

## Results and Discussion

 Drug repositioning or repurposing is an approach successfully used for discovery of new therapeutic purposes for the existing drugs.^18,30-32^ The current study was aimed to use the combination of ligand and structure-based approaches to identify FDA-approved drugs with possible S1P_1_ agonistic activities. S1P_1_ agonists such as siponimod, ozanimod, ponesimod and fingolimod are valuable entities indicated for MS treatment. In addition to these medicines there are also other S1P_1_ agonists which are under investigation in different phases of clinical trials (www.guidetopharmacology.org). In the current study a 3D predictive model for the known 21 S1P_1_ agonists were generated based on 3D-QSAR approach and used to identify FDA-approved drugs with possible S1P_1_ agonistic activity. Then, the selected compounds were screened against S1P_1_ and S1P_3_ receptors by docking calculations to further evaluate their binding abilities and selectivities. Finally, the binding energies of selected compounds were calculated using MD simulations.

###  Generation of 3D-QSAR model for predicting S1P_1 _ agonistic activity

 Generation of predictive models using 3D-QSAR approach for virtual screening has been widely used in drug design and discovery.^19,33-35^ Here, it was intended to employ 3D-QSAR methodology to generate a model for the prediction of S1P_1_ agonistic activity. To this end, the compounds with known activities towards S1P_1_ (Table 1) were docked into S1P_1_ to obtain their receptor bound active conformations. Then, the pose for each compound with the highest score was selected to be used as the train set for the generation of a 3D-QSAR model. The construction of the 3D-QSAR model was carried out using PLS algorithm implemented in Pentacle program and its predictive power was evaluated using LOO and LGO internal cross validation methods. The predicted values for train set compounds using LOO cross validation are available in Table 1 whose correlation with the experimental values was 0.69 with five latent variables (5LVs). Table 2 shows the all statistics regarding the generated model where the calculated r^2^_acc_ for the model was 0.98 with SDEC (standard deviation of error in calculation) value of 0.16 with 5LVs. The LOO q^2^ and LGO q^2^ values for the model with 5LVs were 0.69 and 0.66 with standard deviation error of prediction (SDEP) of 0.68 and 0.71, respectively, indicating the reliability of generated model for prediction of S1P_1_ agonistic activity of potential drug candidates. In the process of development of 3D-QSAR model, the most influential spatial variables, which were common between most of the compounds in train set, were selected for model building. These variables are summarized in Table 3. DRY-DRY (connecting two hydrophobic regions at the distance of 10.8-11.2 Å), N1-N1 (related to two hydrogen binding acceptors positioned 16-16.4 Å apart) and O-N1 (a hydrogen binding donor far apart 12-12.4 from a hydrogen binding acceptor) are three variables whose presence in S1P_1_ agonists favor agonistic activity. These variables showed the highest PLS coefficients in the most active compounds and rarely expressed in the less potent S1P_1_ agonists indicating their applicability for use in building a model for prediction of S1P_1_ agonistic activity. These results as well as the predictivity power assessments support the suitability of the generated model for identifying potential S1P_1_ agonists.

**Table 2:** The statistical data of the built PLS model for S1P_1_ agonists

**No. LVs**	**r**^2^_acc_	**SDEC**	**q**^2^** LOO**	**SDEP**	**q**^2^** RG**	**SDEP**
1	0.69	0.88	0.52	0.85	0.53	0.84
2	0.88	0.42	0.35	0.99	0.50	0.87
3	0.95	0.26	0.50	0.86	0.56	0.81
4	0.97	0.20	0.64	0.73	0.63	0.74
5	0.98	0.16	0.69	0.68	0.66	0.71

Abbreviations: SDEC, standard deviation of error in calculation; SDEP, standard deviation of error of prediction. The acc stands for accumulative value, Validation methods used for calculation of q2 are: leave one out (LOO) and random five groups out (R6GO).

**Table 3:** The most important structural variables in the 3D-QSAR model for S1P_1_ agonists

**Probe block**	**Distance (Å)**	**Impact**
DRY-DRY	10.8-11.2	+
N1-N1	16-16.4	+
O-N1	12-12.4	+
O-TIP	22.8-23.2	-
DRY-N1	2-2.4	-
TIP-TIP	4.4-4.8	-

###  Virtual screening of FDA-approved drugs using the 3D model

**Figure 1:**
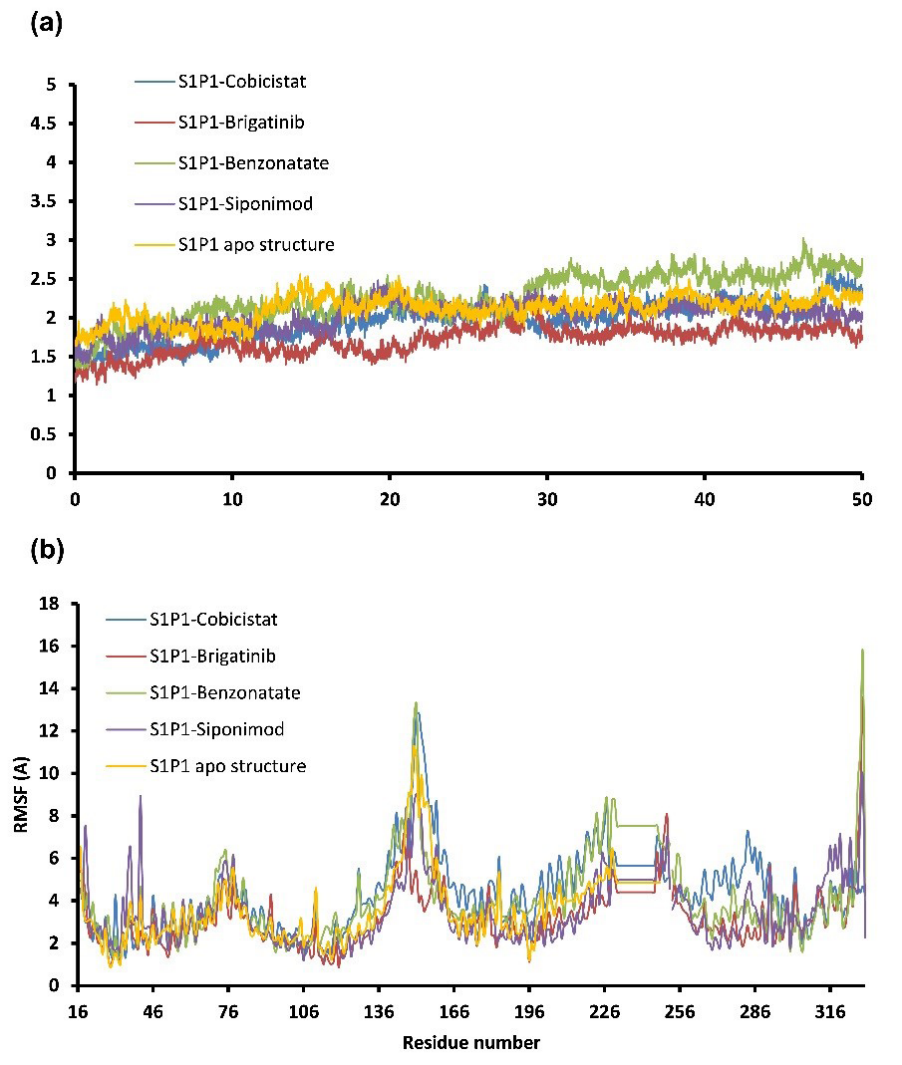
The RMSD (a) and RMSF (per residue RMSD variations) (b) for ligand-S1P_1_ complexes during 50 ns MD simulations

**Figure 2:**
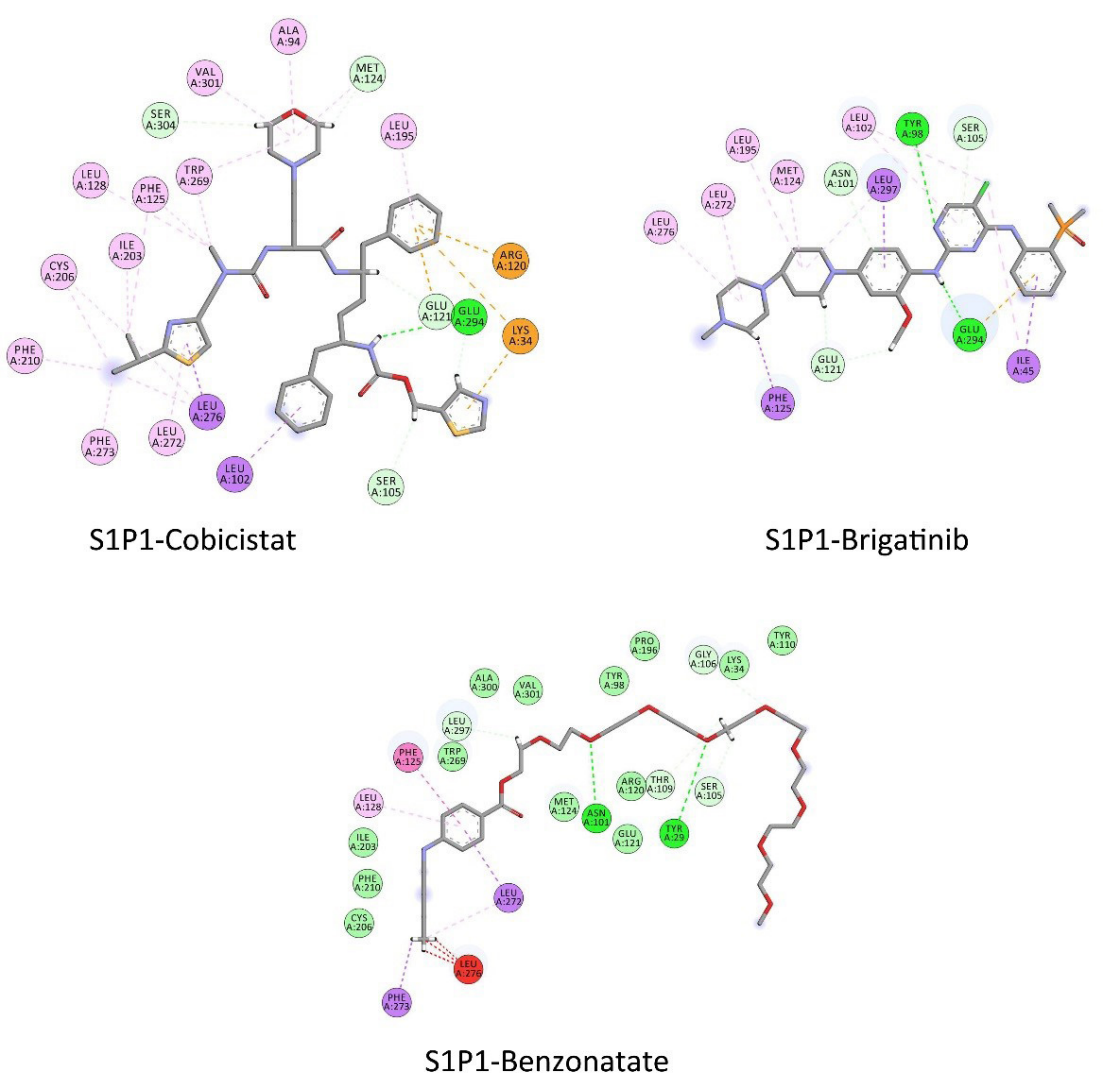
2D-representation of interactions of selected drugs at the binding site of S1P_1_. Discovery Studio Visualization (v 17.2.0) program was used for analysis

**Figure 3:**
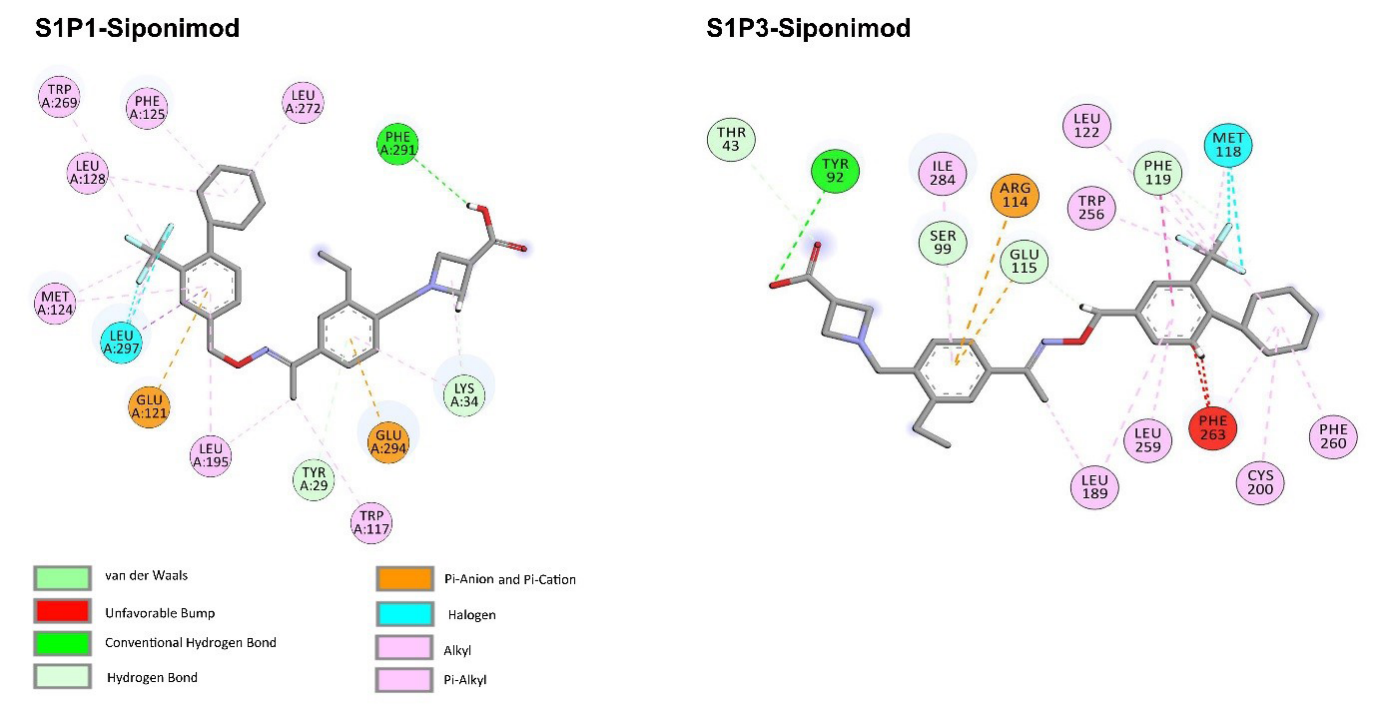
2D-representation of interactions of siponimod at the binding site of S1P_1_ and S1P_3_. Discovery Studio Visualization (v 17.2.0) program was used for analysis

**Figure 4:**
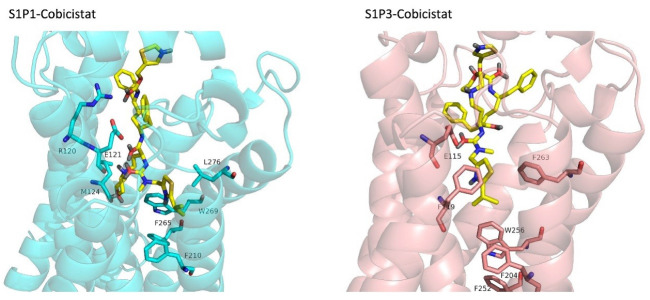
3D-represantation cobicistat docked into S1P_1_ and S1P_3_ binding sites. The receptors are shown in cartoon representations. Cobicistat and binding site residues of receptors are sticks. PyMol program (version 1.7.0.0) was used to generate the images

**Figure 5:**
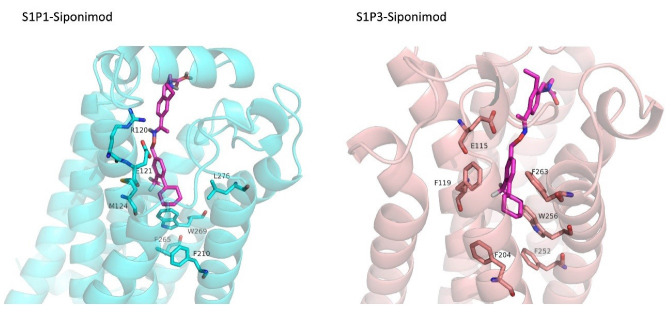
3D-represantation Siponimod docked into S1P_1_ and S1P_3_ binding sites. The receptors are shown in cartoon representations. Siponimod and binding site residues of receptors are sticks. PyMol program (version 1.7.0.0) was used to generate the images

 FDA approved drugs were used as a database for virtual screening to identify potential S1P_1_ agonists using the developed 3D-QSAR model. For this, 1930 drugs deposited until June 11, 2019 (version e-Drug3D_1930) in a databank maintained by Cheminformatic Tools and Databases for Pharmacology (https://chemoinfo.ipmc.cnrs.fr) were used. The S1P_1_ activities of selected solutions were predicted using the previously generated and validated 3D model. Total of 100 drugs with predicted activity greater than that 8.00 (pEC_50_ > 8.00) were selected for further analyses consisting siponimod, an FDA-approved potent S1P_1_, whose predicted pEC_50_ value was 9.4. Among the selected drugs, the procedure resulted in identification of some drugs such as ganirelix and etelcalcetide which showed high predicted S1P_1_ agonistic activities (pEC_50_ values of 18.11 and 17.85, respectively) with negative S1P_1_ docking scores, and hence were regarded as false positives. The identification of false positives can be attributed to the defects of ligand-based drug design methodology in which receptors structure and flexibility as well as solvation effects are not considered.^36^ To prevent these drawbacks, the S1P_1_ docking scores were also considered for screening in addition to the predicted pEC_50_ values. These criteria led to the selection of 20 compounds out of 100 whose predicted pEC_50_ and S1P_1_ docking scores are presented in Table 4. The predicted pEC_50_ and S1P_1_ docking score of siponimod were 9.41 and 90.36, respectively. Therefore, the compounds with the predicted pEC_50_ and S1P_1_ docking score below that of siponimod were eliminated from further study (Table 4). Through this process, the number of compounds for additional study was decreased to 13 (Table 4). Further filtering was carried out by the calculation of S1P_1_ over S1P_3_ selectivity as S1P_1_ selective MS medications show lower adverse effects compared to non selectives.^3^ S1P_3_ model was built based on S1P_1_ crystal structure and evaluated from the geometrical points of view using PROCHECK and MolProbity programs where 100.0% (272/272) of all residues were in allowed ( > 99.8%) regions. The RMSD between the crystal structure of S1P_1_ and the S1P_3_ model was 1.29 Å indicating the appropriate model building using the selected template. The 13 compounds selected based on the predicted pEC_50_ values and screened based on S1P_1_ docking scores (Table 4) were docked into S1P_3_ and among them six compounds with higher S1P1/S1P3 docking scores compared to that of siponimod were selected for MD simulations. By this way, pentagastrin, nilotinib, cobicistat, brigatinib, lapatinib, and benzonatate were considered as the best candidates for further S1P_1_ agonistic activity evaluation. Brigatinib and cobicistat with the S1P_1_/ S1P_3_ ratios of 1.29 and 1.09 were assigned as the most S1P_1_ selective compounds, even better than siponimod with the S1P_1_/ S1P_3_ ratio of 1.02. MD simulations were used to calculate the binding energies of the selected compounds towards S1P_1_. The required topology files for MD simulations were prepared by embedding the complexes of S1P_1_-docked ligands in a lipid bilayer. Simulations were performed for 50 ns and the binding energies for ligand-receptor interaction were calculated using MM-GBSA algorithm based on the entire 50 ns simulations (Table 4). Analyzing the results revealed that cobicistat, benzonatate and brigatinib could bind S1P_1_ with binding energy ΔG° values of -85.93, -69.77 and -67.44 kcal mol ^-1^, respectively, which are greater than the calculated siponimod affinity towards S1P_1_ (-59.35 kcal mol ^-1^). Meanwhile, the affinity of pentagastrin towards S1P_1_ was equal to that of siponimod while lapatinib and nilotinib showed lower affinity to S1P_1_ compared to siponimod (Table 4). Figure 1a shows the RMSD alterations during 50 ns MD simulations on the complexes of cobicistat, benzonatate and brigatinib with S1P_1_, indicating these complexes were structurally stable during 50 ns simulations (Figure 1a). The more precise judgment on RMSD changes upon MD simulations was carried out using RMSF calculation (Figure 1b). Analyzing the results showed that the high RMSF values correspond to unstructured terminal residues and the loop residues linking the third and fourth helices (residues 232 to 246). This loop was missed in crystal structure (PDB ID 3v2y) and modeled using Swiss-Model web server which can be reasoned for residues high fluctuation at this loop during MD simulation. On the other hand, residues located between residue numbers of 136 and 166 showed high fluctuations. However, these fluctuations are not associated with the ligand binding as the S1P1 apo structure also represents the same level of fluctuations during 50 ns MD simulation (Figure 1b). Collectively, from structural point of view, the generated ligand-receptor complexes seem stable during simulations. Cobicistat is an analogue of ritonavir, an anti-HIV medicine, which has no anti-viral effects but is administered by other anti-HIV medicines to inhibit their metabolism by human CYP3A isozyme leading to their increased plasma concentration.^37^ Benzonatate is structurally similar to local anesthetics and exerts its cough relief effects through inhibition of voltage-gated sodium channels and brigatinib is an anaplastic lymphoma kinase inhibitor, a key inducer of non-small cell lung cancer and neuroblastomas. The *in silico* methodologies employed in the current study demonstrated that cobicistat, benzonatate and brigatinib can bind to S1P_1_ with high affinity and exert agonistic activities. Using Discovery Studio Visualization (version 17.2.0) program the possible interactions of cobicistat, benzonatate and brigatinib with S1P_1_ were explored (Figure 2) and compared to that of siponimod (Figure 3). The results indicate that the selected drugs are able to interact with S1P_1_ specific ligand recognition residues, Arg120 and Glu121, located at the extracellular end of third transmembrane helix.^38,39^ Moreover, 2D analyses showed that cobicistat and benzonatate could interact with Phe210 and Trp269 of S1P_1_ whose essential roles to grant agonistic activity have been confirmed by the site-directed mutagenesis studies^40^ supporting that the selected compounds may function as S1P_1_ agonists. Furthermore, the interaction of selected drugs with two selectivity conferring residues (Leu276 and Met124) at the binding site of S1P_1_ was elucidated by 2D analyses, in which brigatinib and cobicistat could establish interactions with Leu276 and Met124, emphasizing the possible selectivity of these compounds towards S1P_1_ rather than S1P_3_ and S1P_4._^40^ More inspection was carried out by comparing the molecular structures of the identified S1P_1_ agonists with that of ML056 in complex with S1P_1_ (PDB ID:3V2Y). ML056 exerts antagonistic activity on S1P_1_ with a phenyl acyl tail inserted into an aromatic pocket of S1P_1_. Hanson et aldemonstrated that extensions applied in the acyl chain can convert the antagonism to agonistic effects due to the increased volume of ligands hydrophobic portion which can lead to establishment of new ligand-receptor interactions.^40^ Such interpretation can be generalized to the identified S1P_1_ agonists in the current study, in which cobicistat with isopropyl thiazolyl (Figure 4), benzonatate with butylamino benzoate and brigatinib with methyl piperazinyl-piperidinyl portions instead of acyl moiety have shown potential agonistic activities. In the current study, the binding mode of siponimod with S1P_1_ and S1P_3_ was explored in order to rationalize its experimentally determined high selectivity towards S1P_1_ rather than S1P_3._^41^ According to the analyses carried out in the current study, siponimod could interact with Glu121 as S1P_1_ recognition residue, Trp269 as agonistic activity granting residue and Met 124 as S1P_1_ selectivity conferring residue (Figure 5). In consistent with the experimental reports,^40,42^ the results showed that while Leu276 in S1P_1_ helps accommodation of siponimod at the binding site, its equivalent residue in S1P_3_, Phe263, narrows down the S1P_3_ ligand binding site, preventing the cyclohexyl-trifluoromethyl phenyl part of siponimod to enter into the binding site (Figure 5). These findings are in agreement with the results of different experimental studies^40,42-45^ supporting the notion that cobicistat, benzonatate and brigatinib may act as S1P_1_ agonists and exert potent and selective pharmacological effects.

**Table 4:** The predicted pEC_50_ values, docking scores and calculated binding energies using MD simulations for siponimod and top 19 compounds selected based on the predicted s1p1 agonistic activities and docking calculations

**Drugs**	**Predicted **S1P_1_**agonistic activity**	S1P_1_**docking score**	S1P_3_**docking score**	S1P_1_**/**S1P_3_** docking scores**	**Binding energy**	**SEM**
Valrubicin	10.91	87.35	-	-	-	-
Pentagastrin	10.90	94.71	89.29	1.06	-60.86	0.08
Nilotinib	12.78	91.09	87.29	1.04	-50.74	0.06
Itraconazole	11.98	74.3	-	-	-	-
Chlorhexidine	12.41	89.81	-	-	-	-
Dabigatran etexilate	11.41	90.33	-	-	-	-
Cobicistat	11.24	103.68	94.74	1.09	**-85.93**	0.13
Paliperidone palmitate	9.73	108.95	123.88	0.88	-	-
Brigatinib	11.21	99.70	77.57	1.29	**-67.44**	0.06
Carfilzomib	10.63	100.01	110.85	0.90	-	-
Lapatinib	10.65	96.64	90.64	1.07	-56.60	0.07
Naloxegol	10.51	82.92	-	-	-	-
Benzonatate	10.41	103.71	97.62	1.06	**-69.77**	0.08
Thonzonium	8.14	104.23	112.72	0.92	**-**	-
Clindamycin palmitate	10.23	99.06	103.99	0.95	-	-
Ritonavir	11.21	93.47	99.03	0.94	-	-
Montelukast	9.93	97.88	107.49	0.91	-	-
Aripiprazole lauroxil	9.91	76.77	-	-	-	-
Lopinavir	9.10	85.64	-	-	-	-
Siponimod	9.41	90.36	88.29	1.02	-59.35	0.05

The calculated binding energies better than that of siponimod are in bold. Prediction of S1P_1_ agonistic activities was performed using the 3D-QSAR model generated in Pentacle program. Docking process was carried out using Gold program and binding energies were calculated using MM-GBSA algorithm implemented in Amber package based on 50 ns molecular dynamics simulation trajectories. The standard errors of means (SEM) were presented for the binding energies.

###  Similarity network analysis

**Figure 6:**
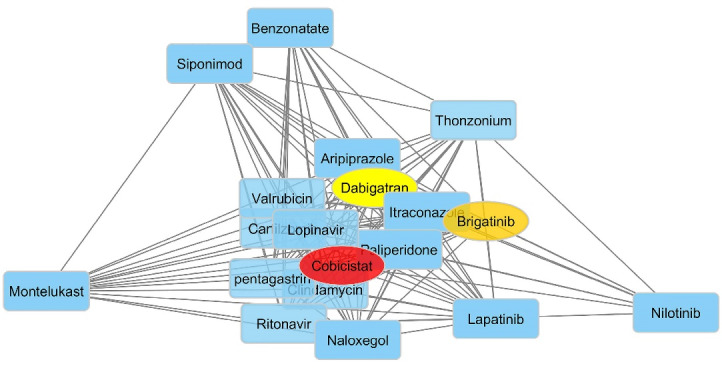
Structure similarity networks for the top 20 S1P_1_ agonists selected based on the predicted pEC_50_ values and docking scores. ChemBioServer was used for network generation. Cytoscape was used to visualize the network

 For the 20 top compounds selected based on the predicted pEC_50_ values and docking scores, the similarity networks were generated. Cytoscape was used to visualize the network in which the hub structures were highlighted based on degree values (Figure 6). The edges weighted based on the similarities between the ligands led to networks in which nodes were clustered into structurally near groups. By looking at the generated network, different groups with diverse chemistry could be noticed which contain different structural backbones such as fatty acid lipids, polypeptides, and phospholipids with distinctive activities including anti-cancer, antiviral, antifungal and leukotriene modifiers. Interestingly, cobicistat was selected as the most structurally connected compound followed by brigatinib and dabigatran. Cobicistat and brigatinib were two drugs which were selected as the potential selective S1P_1_ agonist through exhaustive processes of pEC_50_ Comparing these results with the calculated pEC_50_, docking scores, and binding energies in previous sections indicates that cobicistat and brigatinib possess particular structural features required for conferring S1P_1_ agonistic activity.

## Conclusion

 The current work was aimed to use drug repositioning or repurposing approach for discovery of new therapeutic purposes for the existing drugs by means of *in silico* methods. For this, a 3D-QSAR model was created based on known S1P_1_ agonists and used for virtual screening of FDA-approved drugs. The resultant drugs were filtered based on S1P_1_ and S1P_3_ docking scores and the selected drugs were subjected to MD simulations in order to calculate their binding energies toward S1P_1_. Cobicistat and benzonatate were two drugs which showed superior affinity and selectivity towards S1P_1_ compared to siponimod. Moreover, system analysis also revealed that cobicistat and brigatinib share the highest structural features to the drugs selected by QSAR and docking calculations. The results of this work can be useful for developing novel potent and selective S1P_1_ receptor agonists applicable in MS treatment.

## Acknowledgments

 This work was supported by Research Office of Tabriz University of Medical Sciences [grant number 62634]. The authors would like to thank Biotechnology Research Center of for providing financial and facility supports

## Author Contributions

**Conceptualization: **Ali Akbar Alizadeh.

**Data curation: **Siavoush Dastmalchi.

**Formal Analysis: **Ali Akbar Alizadeh, Behzad Jafari, Siavoush Dastmalchi.

**Funding acquisition: **Ali Akbar Alizadeh.

**Investigation: **Ali Akbar Alizadeh, Behzad Jafari.

**Methodology: **Ali Akbar Alizadeh, Behzad Jafari.

**Supervision: **Siavoush Dastmalchi.

**Validation: **Siavoush Dastmalchi.

**Writing – original draft: **Ali Akbar Alizadeh.

**Writing – review & editing: **Siavoush Dastmalchi.

## Ethical Issues

 Not applicable.

## Conflict of Interest

 The authors declare no conflict of interest.
